# The activity level of follicular helper T cells in the peripheral blood of osteosarcoma patients is associated with poor prognosis

**DOI:** 10.1080/21655979.2022.2031387

**Published:** 2022-01-26

**Authors:** Jianshu Lu, Xiuqin Kang, Zhitao Wang, Gang Zhao, Baoen Jiang

**Affiliations:** Department of Orthopaedics, Dongying People’s Hospital, Dongying, China

**Keywords:** Osteosarcoma, follicular helper T cells, prognosis, overall survival

## Abstract

Osteosarcoma (OS) is solid tumors with high malignancy and incidence starting in the bones. OS pathogenesis has been proved to be closely associated with immune imbalance, and follicular helper T cells (Tfh) significantly affect host humoral immune homeostasis. However, there are few reports on the effect of Tfh cell activation on the prognosis of OS patients. Hence, this investigation on the changes in the proportion of peripheral blood Tfh cells in OS patients, and the relationship between their activity level and OS prognosis. We collected peripheral blood from patients with OS, benign bone tumor (BT group) and healthy subjects (Healthy group), respectively. The number of CD4^+^CXCR5^+^ Tfh cell in peripheral blood was measured by flow cytometry and correlation analysis between its activity and OS clinicopathological characteristics was carried out. The data showed that in comparison with the BT and Healthy groups, higher proportion and activation level of peripheral blood CD4^+^CXCR5^+^ Tfh cells in CD4^+^ T cells were found in the OS group. In OS patients, increases of the proportion and activity level of Tfh cells were associated with poorly differentiated OS and tumor metastasis. Additionally, Kaplan-Meier and Cox regression analysis showed a longer overall survival in patients with low proportion of peripheral blood CD4^+^CXCR5^+^ Tfh cells in CD4^+^ T cells, and their activation level may be a prognostic factor in OS patients. In conclusion, peripheral blood CD4^+^CXCR5^+^ Tfh cell activation in OS patients was associated with a poor prognosis. This study provided ideas for improving the clinical treatment of OS patients.

## Graphical abstract

**Figure:**
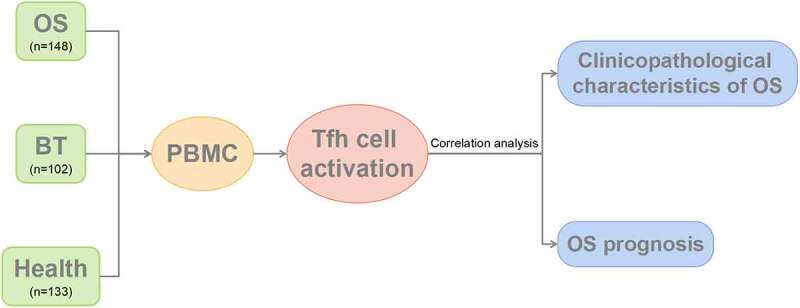

## 1. Introduction

Osteosarcoma (OS) is a highly malignant solid tumor, accounting for about 20% of primary bone malignancies, which originates from mesenchymal tissue and has a predilection for the metaphysics of rapidly growing long bones [1,2]. The incidence of OS is mostly concentrated in the age range of 15–25 years, with a higher incidence in males than in females. OS has a high rate of metastasis and mortality and is the second leading cause of tumor-related death in the adolescent population [3,4]. Additionally, it is characterized by rapid progress, high recurrence rate, and poor prognosis. The 5-year survival rate is only 15%–20% in OS patients who received only single surgery [5]. Amputation was previously the only treatment for bone tumors, but the postoperative survival rate was still as low as 17%, and the basic sports activities and quality of life of patients were greatly affected after surgery [6,7]. With the development of medical level in the past decade, the standardized treatment including surgery and postoperative chemotherapy has achieved a long-term survival rate increasing up to 70% [8]. However, the high cost of treatment and the high disability rate still bring great economic and life burden to patients and society, so it is necessary to study the pathogenesis of OS in order to improve its treatment level [9].

Increasing studies have proved that imbalance of the immune system and tumor immunity is a vital factor leading to the pathogenesis and deterioration of OS [10,11]. Follicular helper T (Tfh) cells, novel T cells discovered recently, have a vital role to play in the regulation of B cell differentiation, antibody production and humoral immunity [12]. Tfh cell is characterized by CXC chemokine receptor 5 (CXCR5) expressing on the cell surface, inducible co-stimulator (ICOS), programed death 1 (PD-1), and IL-21 [13,14]. Tfh regulates the activation of germinal center B cells through surface-expressed CXCR5, PD-1, and ICOS, while Tfh can secrete IL-21 which functions in the production and differentiation of Tfh, germinal center formation, B cell proliferation, and immunoglobulin class switching [15–17]. The involvement of Tfh in the progression of various tumor diseases has been proved, such as peripheral T-cell lymphoma [18]. Current studies have preliminarily confirmed that Tfh cells locally infiltrated in ovarian cancer can mediate humoral immunity by promoting CD8^+^ T cell activity [19]. However, whether the above mechanism is the main mechanism of Tfh cells remains to be confirmed by further studies. Although it has been reported that the ability of Tfh cells to secrete IL-21 is significantly down-regulated in OS patients, thus inhibiting CD8^+^ T cell activation [20,21]. In our previous study, we have demonstrated that microRNA-138 could regulate Tfh cell function and mechanism in OS [22,23]. However, the relationship between Tfh cells and OS clinicopathology has not been studied. Therefore, we detected the activity of Tfh cells in peripheral blood of OS patients with different ages, genders, and tumor stages by flow cytometry. Further, based on the detection results, we explored the relationship between the activity of Tfh cells and the clinicopathological characteristics and prognosis of OS, and then studied the possible role of Tfh in the pathogenesis of OS. This study was carried out with an aim of providing theoretical and experimental basis for the early diagnosis and treatment of OS.

## 2. Materials and methods

### 2.1 Baseline information

Totally 383 subjects were collected from March 2015 to December 2017 in our hospital, including 148 OS patients (OS group), 102 patients with other benign bone tumors (including chondroma, ossifying fibroma, osteochondroma) (BT group), and 133 healthy controls (Healthy group) who underwent physical examination.

All OS patients met the 2002 World Health Organization (WHO) classification. Inclusion criteria were: (1) patients were diagnosed as OS by histological diagnosis [24]: Malignant mesenchymal tumor cells proliferate and produce bone-like and/or bone from these tumor cells; (2) patients did not receive hormone, traditional Chinese medicine or radiotherapy, chemotherapy, and other treatments; (3) patients cooperated with the follow-up survey; (4) patients voluntarily participated and signed the informed consent. Exclusion criteria were: (1) patients with pulmonary nodules or metastatic lesions from other malignant diseases; (2) patients with severe liver and kidney dysfunction and severe infection; (3) patients with a history of other malignant tumors; (4) patients who received glucocorticoid or immunosuppressive drugs in the past three months; (5) patients suffering from autoimmune diseases; (6) patients with low compliance who did not cooperate with follow-up.

**Figure 1.**
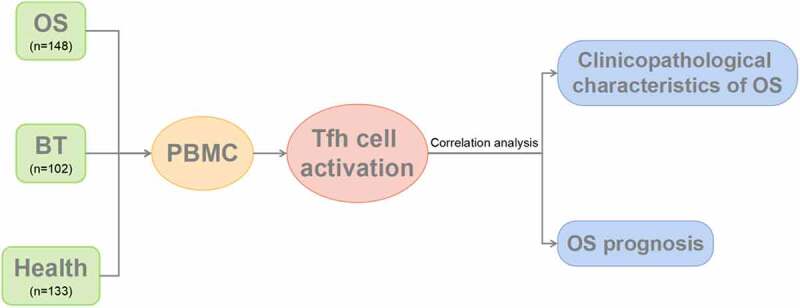
Flow diagram of study design.

OS: osteosarcoma; BT: benign tumor; Healthy: healthy controls; PBMC: peripheral blood mononulcear cell; Tfh: follicular helper T cell.

The research design flow of this experiment was shown in (Figure 1).

### 2.2 PBMCs preparation and Flow cytometric analysis

Totally 5 mL fasting peripheral venous blood sample was drawn and placed into EDTA tubes, followed by gradient centrifugation for isolating peripheral blood mononuclear cells (PBMCs) according to the instructions of the human mononuclear cell separation kit (Solarbio, China). The cell suspension was washed three times in PBS and then PBMCs at 2 × 10^6^/tube were stained with the following monoclonal antibodies: anti-CXCR5-phycoerythrin (PE), anti-CD4-flourescein isothiocyanate (FITC) (BD Bioscience, USA) for 30 min. Then, erythrocyte lysate was added, mixed, and incubated for 20 min away from light. Corresponding isotype control antibody was incubated for 45 min at ambient temperature away from light. Finally, the samples were rinsed by PBS and loaded for detection. According to the related literatures [25,26], T cells with CD4^+^CXCR5^+^ phenotype were defined as Tfh cells. Then, the proportion of CD4^+^CXCR5^+^ Tfh cells and CD4^+^CXCR5^+^CD69^+^ cells in CD4^+^ T cells was measured in PBMCs from the OS, BT, and Healthy groups.

### 2.3 Pathological characteristics and follow-up

The following clinicopathological characteristics of OS patients were recorded, including tumor location, size, stage, differentiation, and metastasis. Follow-up was conducted by telephone and outpatient review, which began when the patient was diagnosed with OS and ended in June 2020. The time from the data of confirmed diagnosis to death or last follow-up is defined as overall survival.

### 2.4 Statistical analysis

All statistical analysis was performed by using SPSS 20.0 (IBM SPSS, Chicago, USA). All data were presented as mean ± standard deviation (SD). The comparison between the two groups was performed using the t-test. A chi-square test was a statistical method adopted for enumeration data. Finally, survival analysis was achieved by Kaplan–Meier method together with the log-rank test. A statistically significant difference was expressed as P < 0.05.

## 3. Results

### 3.1 Baseline information

Clinical characteristics of the three groups are shown in (Table 1). These groups showed no significant difference in age and gender (*P* > 0.05). Then, the OS patients were further grouped according to Tfh cell activity, namely high activity group (High, n = 74) and low activity group (Low, n = 74).

**Table 1.** Comparison of baseline information

Item	OS group (N = 148)	BT group (N = 102)	Healthy group (N = 133)	P value
Age (years), n				0.679
≦20	94	68	91	
>20	54	34	42	
Gender, n				0.878
Male	97	70	89	
Female	51	32	44	
Tumor location, n				
Femur	62			
Tibia	53			
Others	33			
Tumor size (cm), n				
<5	102			
≧5	46			
Tumor stage, n				
IIA	71			
IIB	47			
III	30			
Tumor differentiation, n				
Poorly differentiated	114			
Well-differentiated	34			
Metastasis, n				
Yes	118			
No	30			
Tfh cell activity, n				
Low activity	74			
High activity	74			

OS, Osteosarcoma; BT, benign bone tumor; Tfh, follicular helper T cells.

### 3.2 Changes in the proportion and activity of Tfh cells in peripheral blood of OS patients

**Figure 2.**
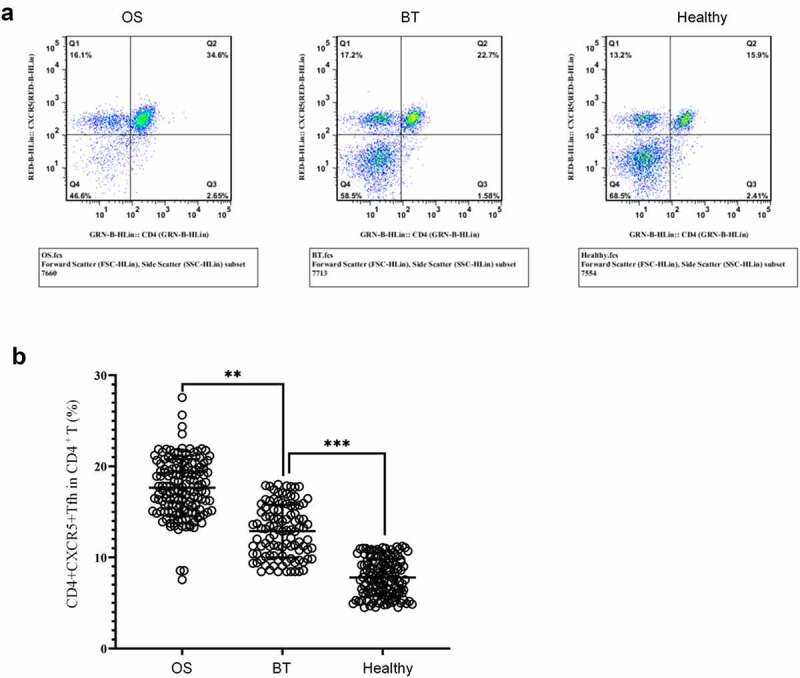
Expression of CD4^+^CXCR5^+^ Tfh cells in peripheral blood mononuclear cells (a) Flow cytometry-based measurement of expression of CD4^+^CXCR5^+^ Tfh cells in peripheral blood mononuclear cell (PBMCs); (b)Proportion of CD4^+^CXCR5^+^ Tfh cells in CD4^+^ T cells. **P < 0.01; ***P < 0.001.

To study the potential role of Tfh cells in the pathogenesis of OS, OS patients, BT patients, and health controls were recruited. Flow cytometry (Figure 2(a,b)) revealed that in comparison with the BT and Healthy groups, the OS group showed a significant higher percentage of CD4^+^CXCR5^+^ Tfh cells in peripheral blood CD4^+^ T cells; in comparison with the Healthy group, this proportion in the BT group was higher.

**Figure 3.**
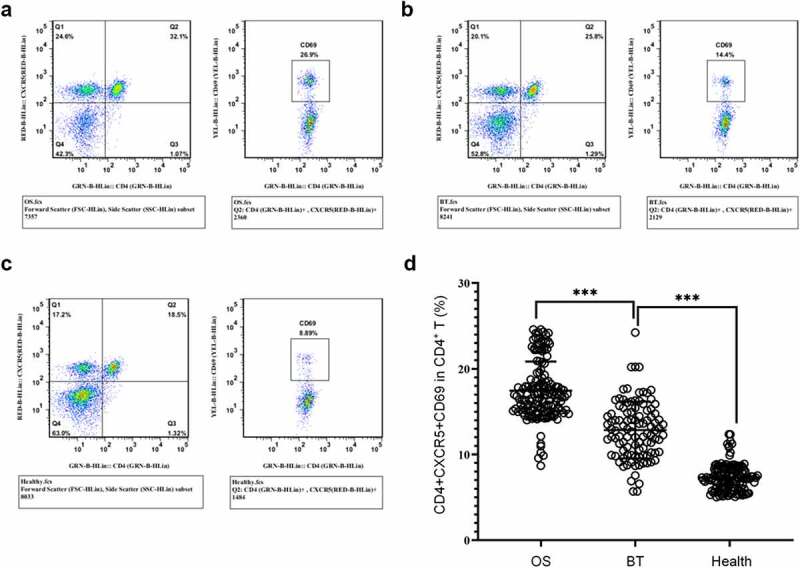
Expression of CD4^+^CXCR5^+^CD69^+^ cells in the peripheral blood (a-c) Flow cytometry-based measurement of expression of CD4^+^CXCR5^+^CD69^+^ cells in peripheral blood mononuclear cell (PBMCs) in the OS group (a), BT group (b), and Healthy group (c); (d) Proportion of CD4^+^CXCR5^+^CD69^+^ cells in CD4^+^ T cells. ***P < 0.001.

It was shown that CD69 is a marker of early activation of T cell subsets [27]. Therefore, we analyzed the frequency of CD69^+^ cells in CD4^+^CXCR5^+^ Tfh cells by FACS. The results showed that OS patients had higher proportion of CD4^+^CXCR5^+^CD69^+^ cells in peripheral blood CD4^+^ T cells in comparison with the other two groups; this proportion in the BT group was increased in comparison with the healthy controls. (Figure 3(a-d)). Collectively, in comparison with the healthy control, a marked increase was identified in the proportion and activity level of CD4^+^CXCR5^+^ Tfh cells in PBMCs of OS cases.

### 3.3 Correlation analysis between the activity level of Tfh cells and clinicopathological characteristics of OS

**Figure 4.**
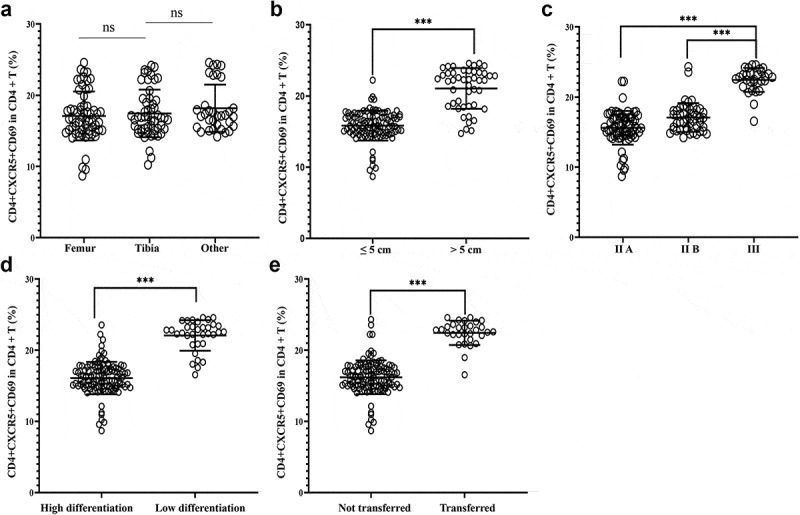
Correlation analysis between the activity level of Tfh cells and clinicopathological characteristics of osteosarcoma. (a) Tumor location; (b) Tumor size; (c) Tumor stage; (d) Tumor differentiation; (e) Presence or absence of distant metastasis. ***P < 0.001; ns, no significant.

In addition, an analysis was carried out on the association between the Tfh cell activity and OS clinicopathological characteristics. The results revealed that the proportion of CD4^+^CXCR5^+^CD69^+^ cells in peripheral blood CD4^+^ T cells was independent of tumor location in OS patients (Figure 4(a)). However, in comparison with OS patients with tumor size < 5 cm, this proportion was higher when tumor size ≥ 5 cm (Figure 4(b)); in comparison with OS patients with tumor stages IIA and IIB, this proportion was higher in tumor stage III (Figure 4(c)); in comparison with poorly differentiated OS, this proportion showed an increase in well-differentiated OS (Figure 4(d)); in comparison with OS patients without tumor metastasis, a marked increase of this proportion was identified in patients with tumor metastasis (Figure 4(e)).

### 3.4 Survival analysis between Tfh cell activity level and OS prognosis

**Figure 5.**
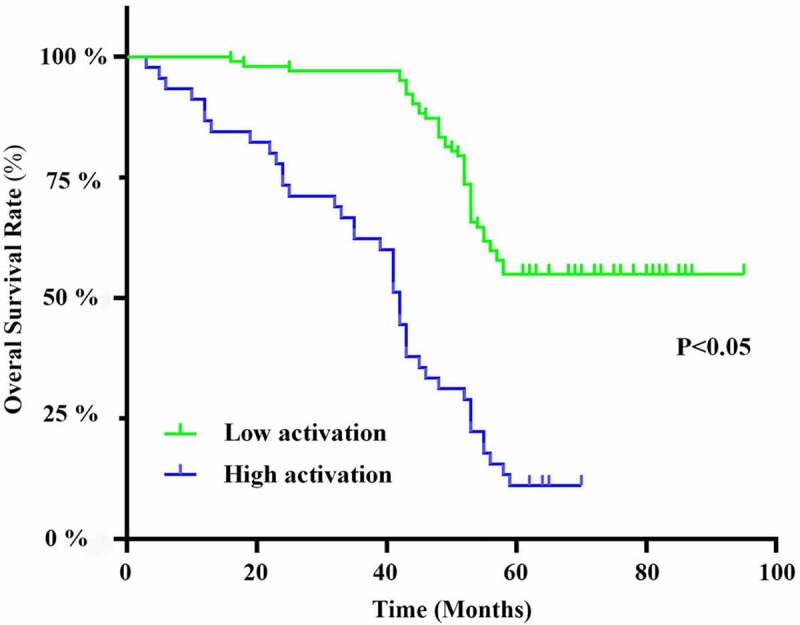
Kaplan-Meier survival curve. Overall survival of osteosarcoma patients with high versus low activation levels of Tfh cells in peripheral blood.

On the basis of the median value of the frequency ratio of circulating Tfh cells in peripheral blood, OS cases grouped into 74 cases of low Tfh cell activity and 74 cases of high activity. Kaplan-Meier analysis (Figure 5) showed a longer overall survival in patients with a low proportion of CD4^+^CXCR5^+^CD69^+^ cells in peripheral blood.

Further univariate results as well as multivariate regression analysis proved that tumor size, tumor stage, tumor differentiation, presence of metastasis, and Tfh cell activation level could be factors related to the OS prognosis, while tumor location was not an indicator of OS prognosis (Table 2).

**Table 2.** Univariate and multivariate Cox analysis of overall survival in osteosarcoma patients

Item	Type	Univariate		Multivariate	
		HR (95%CI)	P	HR (95%CI)	P
Tumor location	Femur/Tibia/ Others	0.998 (0.655–1.521)	0.994	1.036 (0.778–1.379)	0.809
Tumor size	≦5 cm/>5 cm	5.245 (2.227–12.352)	<0.001	0.708 (0.349–1.435)	0.338
Tumor stage	IIA/IIB/III	6.636 (3.436–12.814)	<0.001	4.034 (2.282–7.132)	**<0.001**
Tumor differentiation	Poorly differentiated/ well-differentiated	10.897 (3.150–37.699)	<0.001	2.071 (1.066–4.023)	**0.032**
Tumor metastasis	With/without metastasis	8.847 (2.543–30.777)	0.001	0.236 (0.085–0.655)	**0.006**
Tfh activity level	Low/high activation	9.739 (3.554–26.691)	<0.001	2.280 (1.202–4.325)	**0.012**

OS, Osteosarcoma; BT, benign bone tumor; Tfh, follicular helper T cells.

## 4. Discussion

OS contributes the majority of clinically malignant bone tumor diseases in adolescents. However, its pathogenesis is still unclear and its pathogenic factors are complex, including genetic factors, environmental factors, and external causes such as chemicals substances and internal and external irradiation, and chronic inflammatory stimulation [28]. At present, a comprehensive treatment based on surgery and adjuvant chemotherapy is adopted for treating OS. Significant differences are found in the treatment levels of OS among different medical institutions in China, and amputation is still the main treatment for OS of the limb in some remote areas [29]. In recent years, many scholars have found that imbalance of immune status and alteration of tumor immune status play an important role in the OS pathogenesis as well as tumor proliferation [30]. In the complex OS microenvironment, T lymphocytes significantly affect the process of tumor immunity [31], and Tumor immune-based treatment is expected to be a breakthrough point for OS treatment.

Tfh cells is a subset of CD4^+^ T cells regulating B cell differentiation, antibody secretion, and humoral immunity. Tfh cells are characterized by surface expression of CXCR5, PD-1, and ICOS, and activate germinal center B cells with the help of these surface markers [32,33]. Some researchers have found that Tfh cells are involved in and functions in immune diseases such as rheumatoid arthritis [34]. It has also been reported that Tfh plays an important role in the development of various oncological diseases such as peripheral T-cell lymphoma and follicular lymphoma [35–37]. In this study, we examined the proportion and activity level of peripheral blood Tfh cells in OS cases with different ages, stages, and genders. Based on the detection results, we deeply analyzed the relationship between Tfh cell activity and the prognosis of OS patients. Specifically, in comparison with the BT and Healthy groups, the OS group showed a significant higher proportion and cell activity of Tfh cells, suggesting that the humoral immunity of OS patients was inhibited. We also found that with the increase of OS stages, the proportion and activity level of peripheral blood Tfh cells were increased, but no significant difference was identified.

There is still no relevant study on the relationship between the proportion and activity level of Tfh cells and OS. In this study, Kaplan–Meier analysis revealed a longer survival in OS patients with a low proportion of peripheral blood CD4^+^CXCR5^+^CD69^+^ cells, suggesting that this index may have some predictive value for prognosis. Although previous studies have reported an upregulation in the frequency of peripheral blood Tfh cells of OS patients [38], the proportion and activity level of Tfh cells have not been detected simultaneously. Current studies have confirmed that compared with the single detection of Tfh cell frequency, the combined detection of the proportion and activity level of Tfh cells can more accurately reflect the changes in the intensity of host humoral immunity [39]. In this study, we also systematically analyzed the survival status of OS based on Tfh cell activation, and confirmed that Tfh cell activation can be a relevant factor for the prognosis of OS patients using Cox regression analysis. Our findings provide more detailed and accurate evidence for a comprehensive understanding of what relationship Tfh cells have with OS.

Role and mechanism of Tfh cells in tumor diseases are still unclear, although numerous studies have found that the number of Tfh cell is markedly elevated in various tumor diseases [40,41]. Down-regulation of IL-21 secretion capacity of Tfh cells has reported in OS patients, resulting in the inhibition of CD8^+^ T cell activation [20]. However, the other mechanisms and the specific mechanism of Tfh cells in OS progression remain to be further studied.

## 5. Conclusions

In summary, we found that the percentage and activity level of peripheral blood Tfh cells were higher in patients with OS and were closely associated with poorly differentiated osteosarcoma and tumor metastasis. The proportion of Tfh cells with high activation levels showed a worse prognosis. These results contribute to our understanding of the pathogenesis of OS. However, due to the limited sample size, the results of this study still need to be supported by data from large sample multicenter trials.

## Supplementary Material

Supplemental Material

## Highlights

1. The proportion of Tfh cells was significantly elevated in the peripheral blood of patients with osteosarcoma.
2. Increases of the proportion and activity level of Tfh cells were associated with poorly differentiated osteosarcoma and tumor metastasis.
3. Cell activation in osteosarcoma patients was associated with a poor prognosis.
